# Legionella: Organizing Pneumonia vs Persistent Infection

**DOI:** 10.7759/cureus.29685

**Published:** 2022-09-28

**Authors:** Ayham Khrais, Brian Smighelschi, Maliha Zainab

**Affiliations:** 1 Department of Medicine, Rutgers University New Jersey Medical School, Newark, USA

**Keywords:** nonspecific interstitial lung disease (nsip), legionnaire’s disease, pneumonia, organizing pneumonia, legionella

## Abstract

Organizing pneumonia is a rare subtype of interstitial lung disease that can occur following infectious alveolar insults. Imaging often demonstrates bilateral patchy opacification while biopsy reveals irregular alveolar fibrosis. Steroids are the treatment of choice, resulting in rapid clinical improvement. In this report, we describe a 69-year-old woman with a recent hospitalization for *Legionella* pneumonia who presented with worsening dyspnea and radiographic evidence of bilateral patchy infiltrates. Differential diagnoses included *Legionella *treatment failure and organizing pneumonia, therefore she was managed with both antibiotics and steroids. Her clinical status improved significantly the day after treatment initiation, making organizing pneumonia as the likely culprit for her initial decompensation. In patients with a recent history of lung injury who present with acute hypoxic respiratory failure, one must have a high index of suspicion for organizing pneumonia, for prompt treatment often results in rapid recovery.

## Introduction

Interstitial lung disease describes a wide spectrum of restrictive lung injury, with different subtypes often identified by histology. Organizing pneumonia is an acute form of interstitial lung disease describing irregular deposition of fibrin within alveoli [1,2]. This development of patchy lung fibrosis is rare and can develop following an insult to the alveoli, including various types of pathogen-associated pneumonia, connective tissue diseases, and medications. Treatment involves systemic steroids, which can result in rapid symptom and radiographic improvement [2]. Here we describe an interesting case of organizing pneumonia that developed in a patient who had previously undergone a prolonged hospitalization for *Legionella *pneumonia.

## Case presentation

A 69-year-old woman with a history of hypertension and tobacco use disorder presented from subacute rehabilitation with shortness of breath (SOB) after a chest X-ray (CXR) demonstrated bilateral lung infiltrates. The patient’s history of tobacco use consisted of smoking one pack of cigarettes per day for over 30 years. Of note, the patient was hospitalized approximately 3 weeks prior for acute hypoxic respiratory failure secondary to *Legionella *pneumonia, with a hospital course complicated by septic shock requiring bilevel positive airway pressure (BiPAP) and intravenous fluids. At that time, she was treated with a total 10-day course of levofloxacin and discharged on home oxygen via nasal cannula. Since that hospitalization, her SOB was mild but persistent. Five days prior to this current admission, her SOB acutely worsened, requiring up-titration of her oxygen from a baseline of 2 L via nasal cannula up to 4 L, associated with a cough productive of tan sputum and subjective fevers. CXR there was significant for right middle lobe and superior left lower lobe infiltrates, therefore the patient was brought to the emergency department (ED) for further evaluation.

**Figure 1:**
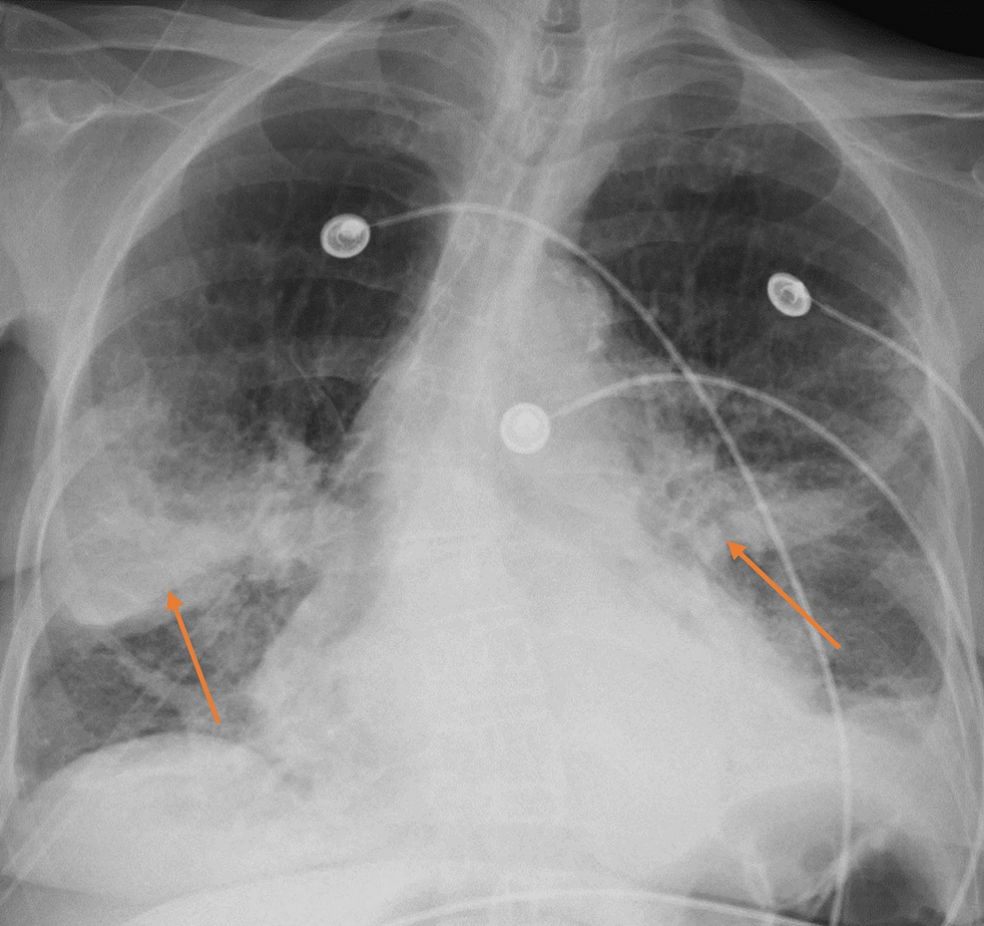
Chest X-Ray Demonstrating Right Middle Lobe and Superior Left Lower Lobe Infiltrates (arrows).

In the ED, patient’s vitals were significant with a blood pressure of 131/75 mmHg, heart rate of 129 beats per minute, temperature of 100.8°F, and oxygen saturation of 87% on room air. Physical exam was notable for diffuse bilateral biphasic rhonchi and end-expiratory wheezes. Laboratory evaluation demonstrated potassium of 3.2 mEq/L and magnesium of 1.5 mEq/L without leukocytosis. CXR in the ED was notable for opacities similar to those found on the CXR (Figure 1). The patient was placed on high-flow nasal cannula at 50 L per minute at a FiO_2_ of 100% with good subsequent oxygen saturation. She was given cefepime and azithromycin and admitted for management of acute hypoxic respiratory failure.

**Figure 2:**
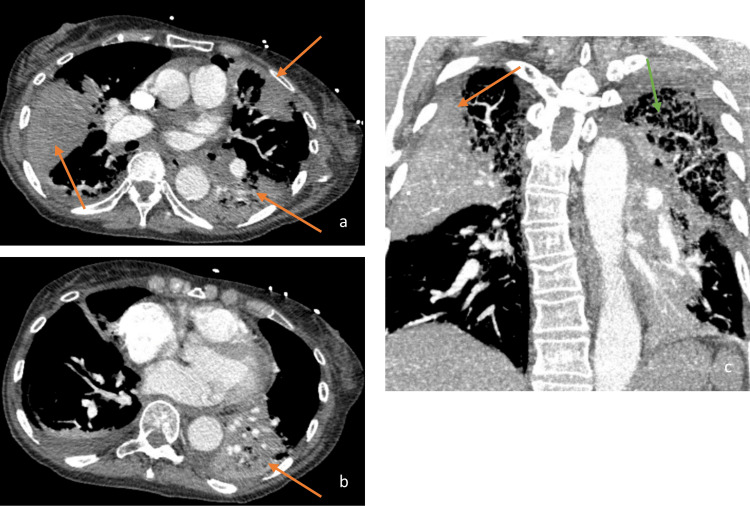
Transverse (a, b) and Coronal (c) Views of a Computed Tomography Angiography Scan of the Chest. The Scan Demonstrating Multifocal Opacities Within the Right Middle & Superior Left Lower Lobes of the Lungs (orange arrows) and Emphysematous Changes (green arrow).

**Figure 3:**
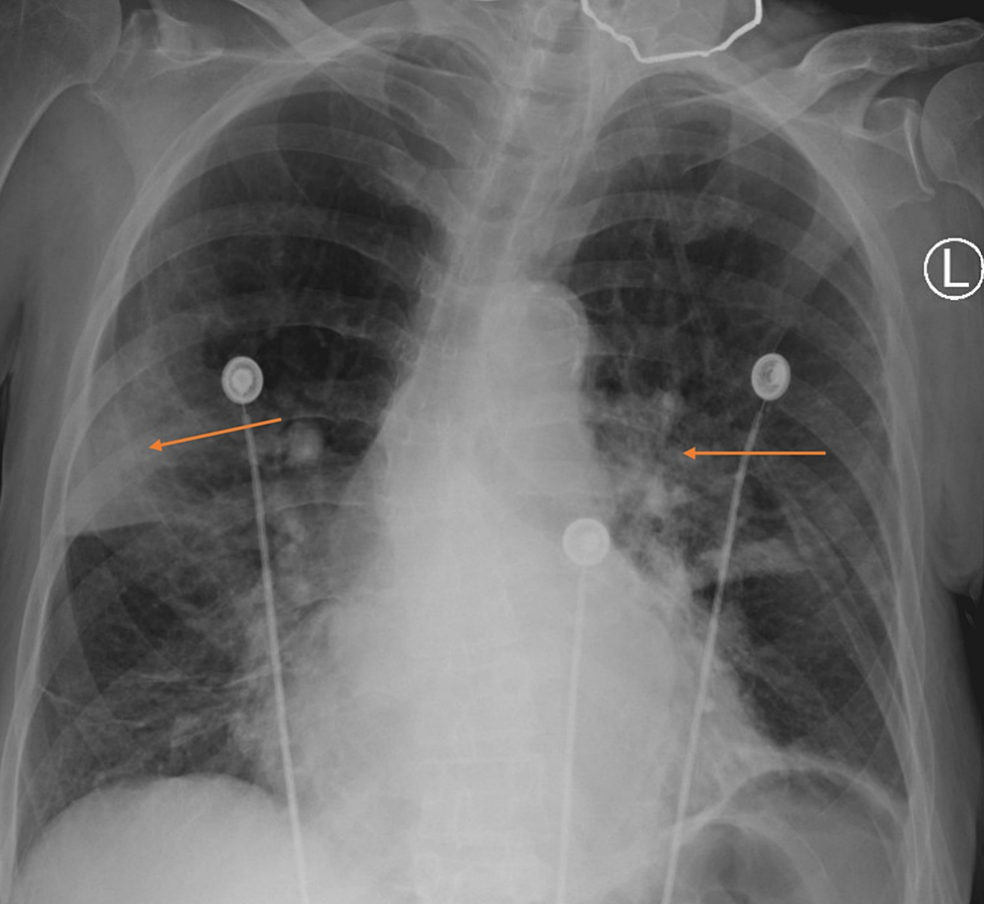
Repeat Chest X-Ray Demonstrating Improvement in Bilateral Lung Opacification (arrows).

The patient was started on broad-spectrum antibiotics for possible hospital-acquired pneumonia, including vancomycin and cefepime. Persistent bilateral diffuse end-expiratory wheezing and biphasic rhonchi were noted on lung auscultation. Computed tomographic angiography (CTA) of the chest, to rule out pulmonary embolism (PE), was performed demonstrating emphysema and multifocal opacifications (Figure 2). No PE was seen on CTA. The patient was started on standing ipratropium-albuterol. The urine *Legionella *antigen test was positive. Infectious disease and pulmonology were consulted. As the *Legionella *urine test can be positive for months after infection and the patient remained afebrile without leukocytosis, the patient was believed to have organizing pneumonia as a sequela of prior *Legionella *infection. Despite this, she was maintained on levofloxacin for possible treatment-resistant *Legionella *pneumonia, and steroids were started for organizing pneumonia with concomitant trimethoprim-sulfamethoxazole for Pneumocystis prophylaxis. Respiratory status improved significantly one day after treatment initiation, with the patient demonstrating no accessory respiratory muscle use and saturating well on 3 L of oxygen via nasal cannula. Repeat CXR showed improvement in previously noted opacities (Figure 3). The patient was discharged in stable medical condition with outpatient pulmonology follow-up, to complete a 6-12 week course of steroids.

## Discussion

Legionnaires’ disease describes pneumonia caused by the *Legionella* species, with the most common and well-documented pathogen being *Legionella pneumophila*. *Legionella *species are gram-negative and found in freshwater environments, so transmission occurs with inhalation of aerosols or aspiration of water-containing *Legionella *species [3,4]. In 2012, Legionnaires’ disease was reported at a rate of 10.8 per 1 million people in the United States, 72% of which were in patients greater than 50 years old [4]. If left untreated, this systemic infection has high mortality and morbidity [4]. Legionnaires’ has a wide range of clinical manifestations, but can include fever, non-productive cough, headache, myalgias, rigors, dyspnea, diarrhea, and/or delirium [3]. While alveolar infiltrates on CXR are more common in Legionnaires’ disease, there is no CXR pattern that separates this infection from other forms of pneumonia [3]. Of the modalities available to diagnose Legionnaires’, the tests of choice are sputum culture on a *Legionella*-specific medium, urinary antigen detection, and nucleic acid amplification [5].

As it is difficult to differentiate Legionnaires’ disease from other causes of community-acquired pneumonia, it is important that initial antibiotic management includes an agent effective against *Legionella *species [4]. Unlike the other causative organisms for community-acquired pneumonia which can be treated with a beta-lactamase, treatment of *Legionella *requires the addition of an antibiotic that achieves therapeutic levels within alveolar macrophages given its intracellular pathogenesis [4]. These antibiotics include macrolides, fluoroquinolones, and tetracyclines. According to Infectious Diseases Society of America (IDSA)/the American Thoracic Society (ATS) guidelines, patients with Legionnaires’ disease should receive monotherapy with one of the mentioned antibiotics for 5-12 days and should not be stopped until the patient is afebrile for 48-72 hours [4]. This therapy can be extended for up to 21 days if the patient is immunocompromised, or longer if complications arise [4]. It has also been found that combination therapy for the treatment of Legionnaires’ disease has no straightforward evidence showing superiority, even in severe cases [4].

In this patient whose condition was refractory to appropriate management with the initial diagnosis of *Legionella*, the differential diagnosis was broadened to include organizing pneumonia. Patients with organizing pneumonia typically present nonspecifically at first, but progressively develop a mild fever, cough, malaise, anorexia, weight loss, and dyspnea [6]. Although most present as mild dyspnea, those with rapid, progressive disease, which typically develops over a few weeks, can develop severe dyspnea [6]. Causative agents for organizing pneumonia include bacteria such as *Streptococcus pneumoniae *and *L. pneumophila*, viruses, *Plasmodium vivax*, fungi such as *Pneumocystis jiroveci*, and medications [6]. The most common demonstration on CT of the chest would show multiple patchy alveolar opacities, with densities ranging from ground-glass to consolidation, and air bronchograms [6,7]. The pathogenesis of organizing pneumonia can be broken down into three stages: the early injury phase, the proliferating phase, and the mature phase [6]. The injury phase consists of plasma proteins infiltrating the alveolar lumen, activation of the coagulation cascade with fibrin deposits, and migration of inflammatory cells [6]. The proliferating phase is carried out through the breakdown of fibrin leading to the migration of activated fibroblasts, resulting in proliferation and differentiation into myofibroblasts [6]. This swarm of myofibroblasts forms cell clusters within the distal airspaces, replacing the fibrin with loose connective tissue rich in collagen [6]. The final, mature phase is characterized by concentric rings of fibrotic buds consisting of myofibroblasts interlaced with alternating layers of collagen clearly delineated in the air space [6].

In the absence of a confirmatory lung biopsy demonstrating this classical pathological pattern, organizing pneumonia cannot be diagnosed with certainty and instead would require strong clinical suspicion and close observation with the initiation of treatment. The standard therapy for organizing pneumonia is corticosteroids and, if the diagnosis is correct, would result in the regression of symptoms in days, as was seen in this patient [6]. Furthermore, with treatment, radiographic findings improve rapidly, as was also observed in this case [6]. There is no established dose or length of corticosteroid therapy, but in practice, patients have been given 0.75 to 1.25 mg/kg/day of prednisolone tapered over 12-22 weeks [6]. Augmentation with clarithromycin has shown to be beneficial in reducing the duration of the corticosteroid regimen [6]. In patients with persistent disease, the addition of immunosuppressive therapy using cyclophosphamide or azathioprine is warranted, with the corticosteroid dose lowered to approximately 0.25 mg/kg/day [6]. Relapses, which occur in upwards of 58% of patients, are not associated with increased morbidity or mortality and quickly resolve with resumed therapy, using 20 mg of prednisone daily [6]. The prognosis for patients with organizing pneumonia is excellent [6].

Few cases of *Legionella*-associated organizing pneumonia are reported in the literature. One such case reports persistence of symptoms despite antibiotic therapy, with rapid resolution one day after initiation of steroids [8]. This rapid amelioration of symptoms is similar to that observed in the patient we discussed. However, the reported case involved a definitive diagnosis of organizing pneumonia via biopsy, while such invasive measures were deferred in our patient given rapid symptom improvement [8]. We report a similar case of initially suspected *Legionella *infection refractory to steroids, but with significant recovery after corticosteroid initiation. Therefore, one must have a high suspicion of organizing pneumonia in a patient with a history of *Legionella *infection and positive *Legionella *testing presenting with an SOB that is refractory to antibiotic treatment.

## Conclusions

Here we describe a likely case of *Legionella*-induced organizing pneumonia, as evidenced by worsening hypoxia in a patient who recently completed adequate treatment for *Legionella *pneumonia. Our patient’s symptoms and radiographic findings improved rapidly after the initiation of steroids, further supporting the diagnosis of organizing pneumonia as opposed to infectious pneumonia. *Legionella*-associated organizing pneumonia must be considered in the differential of patients who develop respiratory decompensation a few weeks after being treated for *Legionella *infection, for this condition is easily treated and has a good prognosis.
